# The comprehensive complication index is more sensitive than the Clavien–Dindo classification for grading complications in elderly patients after radical cystectomy and pelvic lymph node dissection: Implementing the European Association of Urology guideline

**DOI:** 10.3389/fonc.2022.1002110

**Published:** 2022-10-20

**Authors:** Haiwen Huang, Zhenan Zhang, Han Hao, Haixin Wang, Meixia Shang, Zhijun Xi

**Affiliations:** ^1^ Department of Urology, Peking University First Hospital, Beijing, China; ^2^ Institute of Urology, Peking University, National Urological Cancer Center, Beijing, China; ^3^ Institute of Urology, National Research Center for Genitourinary Oncology, Beijing, China; ^4^ Department of Urology, Yankuang New Journey General Hospital, Zoucheng, China; ^5^ Department of Medical Statistics, Peking University First Hospital, Beijing, China

**Keywords:** elderly patient, radical cystectomy, complications, EAU guidelines, comprehensive complication index

## Abstract

**Objectives:**

Lack of assessment of 90-d perioperative morbidity in elderly patients after radical cystectomy and pelvic lymph node dissection (PLND) using a standard reporting methodology, and the Clavien–Dindo classification (CDC) does not accurately reflect the burden of complications. We aim to report the 90-d complications of elderly patients after radical cystectomy, and to compare the validity of the Comprehensive Complication Index (CCI) and CDC.

**Methods:**

Retrospective review of 280 patients aged ≥75 years who received radical cystectomy between 2006 and 2021. The 90-d complications of elderly patients after radical cystectomy were reported by implementing the EAU criteria. The CDC and CCI were both used for grading complications. The Spearman rank correlation coefficient was used to estimate the correlation between postoperative stay and CDC/CCI. Logistic regression was used to identify the risk factors for major complications. The sample size for a fictive superiority trial was calculated for different endpoints.

**Results:**

A total of 225 (80.36%) patients suffered from 528 complications. The cumulative CCI had a more accurate prediction of postoperative stay than the CDC (r = 0.378, p < 0.001 vs. r = 0.349, p < 0.001). The need for sample size could decrease when CCI was used for the primary endpoint. More risk factors for major complications were identified when CCI ≥33.7 was defined as the endpoint of major complications.

**Conclusion:**

CCI is better than CDC for grading the severity of complications in elderly patients after radical cystectomy and PLND.

## Introduction

Radical cystectomy is recommended by evidenced-based guidelines as the gold standard treatment for patients with recurrent high-grade non-muscle-invasive bladder cancer and local muscle-invasive bladder cancer (1). Because of its severe trauma and high complexity, patients are susceptible to various complications after operations. However, the incidence of complications after radical cystectomy varied widely in different studies (2–7). An unclear definition of complications and inconsistent reporting criteria account for this difference. The European Association of Urology (EAU) has published a guideline of complication reporting for urological surgical procedures (8), which has been validated in open radical cystectomy and robot-assisted radical prostatectomy (9, 10). Nonetheless, no study has yet assessed the 90-d perioperative morbidity of elderly patients after radical cystectomy and pelvic lymph node dissection (PLND) using a standard reporting methodology.

The Clavien–Dindo classification (CDC) was recommended as a severity grading system for postoperative complications (8). However, only the most serious complication was considered for grading when using CDC (11). Patients after radical cystectomy often suffer from more than one complication, and the main limitation of CDC is the underestimation of the burden of perioperative complications. To estimate the burden of complications more accurately, the Comprehensive Complication Index (CCI) was proposed by Slankamenac and colleagues (12), which based the complication grading by CDC and considered all complications into a single formula weighted by their severity. The CCI has been validated for open radical cystectomy in a previous study (10, 13). The incidence of bladder cancer increases with age, and most patients are elderly (14). Elderly patients often have more comorbidities, which leads to a higher risk of complications. We hypothesized that the burden of complications would be estimated more accurately when CCI was used.

The aims of the present study are threefold: (1) to assess the 90-d complications of elderly patients after radical cystectomy and PLND based on EAU guideline of complication reporting, (2) to compare CCI with CDC in elderly patients, and (3) to explore the risks of major complications after operations according to CDC and CCI.

## Materials and methods

### Patient population

Retrospective analysis of data from chart review was performed in this study. We defined the following inclusion criteria: (1) patients who received radical cystectomy with urinary diversion from January 2006 to October 2021 at Peking University First Hospital and (2) patients aged ≥75 years. The exclusion criteria were as follows: (1) PLND was not performed (n = 130) and (2) patients received neoadjuvant chemotherapy or radiotherapy before surgery (n = 4). Finally, a total of 280 consecutive patients were enrolled in this study. Ethical approval for this study was obtained from the clinical research ethics committee of Peking University First Hospital, Beijing, China.

### Assessment of complications

The collection and grading of complications were completed by two clinical doctors. Defines of complications after radical cystectomy were referred to the catalog of study of Vetterlein, Malte W. et al (10), which employed the Common Terminology Criteria for Adverse Events (CTCAE) v5.0. The 90-d complications after radical cystectomy were collected from digitalized charts, and outpatient information was included. Each complication was graded by using the CDC (11), and the highest grade for each patient was defined as the CDC of the patient. The CCI was calculated for each patient by using the online tool provided at https://www.assessurgery.com. The cumulative CCI and non-cumulative CCI were calculated based on all complications and the highest complication, respectively. The intraoperative complications were reported separately. This study complied with the EAU guideline of complication reporting (**Supplementary Table 1**).

### Clinical characteristics and pathological data

Clinical characteristics, including sex, age, BMI, type of surgical approach, type of urinary diversion, time of operation, preoperative and postoperative hemoglobin, estimated blood loss (EBL), and postoperative stay, were collected in this study. The Charlson comorbidity index was used to assess the comorbidity of patients (15). The last recorded preoperative serum creatinine (sCr) values were extracted, and sCr ≥ 1.5 mg/dL was classified as renal insufficiency. Pathological T stage and pathological nodal stage were classified by using the TNM staging system of bladder cancer of the American Joint Committee on Cancer Staging Manual 8th edition. Furthermore, pathological grade, lymph node yield, and surgical margin status were included in the pathological data.

### Statistical analysis

The Spearman rank correlation coefficient was used to estimate the correlation between variables. Logistic regression was used to identify the risk factors for major complications for all patients, which included the variables that showed a univariate relationship with major complications or that were considered clinically relevant. The sample size for a fictive superiority trial was calculated when the presence of any CDC complication, the presence of a major CDC complication, or the cumulative CCI were used as the primary endpoints. We assume a 30% relative risk reduction for the incidence of any complication and the incidence of major complications, a difference of 10 points for the cumulative CCI, a power of 80%, and an α-error of 0.05. A difference of 10 points on the cumulative CCI reflects one grade difference of CDC (12). The statistical analyses were performed using IBM SPSS version 25 (IBM Corporation, Armonk, NY, USA), and all *p* values were two-sided. *p* < 0.05 was considered to be statistically significant. R (version 3.6.3) was used for visualization of correlation analyses, and Prism 6.0 (GraphPad, Prism^®^) was used for graphing.

## Result

### Clinical and pathological characteristics

A total of 280 elderly patients who underwent radical cystectomy and PLND were enrolled in this study. The clinical and pathological characteristics are depicted in **Table 1**. Among them, 169 (60.4%) patients received open radical cystectomy, 99 (35.4%) patients underwent laparoscopic radical cystectomy, and 12 (4.43%) patients received robot-assisted radical cystectomy. The median age of all patients was 78 (76-80) years, and the median postoperative stay was 10 (8-14) days.

**Table 1:** The clinicopathologic characteristics of all patients.

	Overall (n = 280)
Male	228 (81.4%)
Age	78 (76-80)
BMI (kg/m^2^)	23.77 ± 3.39
Charlson comorbidity index	
0	137 (48.9%)
1-2	112 (40.0%)
3-4	27 (9.6%)
≥5	4 (1.4%)
ORC	169 (60.4%)
LRC	99 (35.4%)
RARC	12 (4.43%)
Type of urinary diversion	
ureterocutaneostomy	174 (62.1%)
Ileal conduit	103 (36.8%)
Orthotopic neobladder	3 (1.1%)
Preoperative renal insufficiency	31 (11.1%)
Time of operation (mins)	223 (172-294.5)
EBL (L)	300 (125-600)
Postoperative stay (days)	10 (8-14)
ΔHb	22.22 (15.71-29.15)
Pathological T stage	
Ta and Tis and T1	80 (28.6%)
T2	86 (3.7%)
T3	70 (25.0%)
T4	44 (15.7%)
Pathological nodal stage	
N0	239 (85.4%)
N1	14 (5.0%)
N2	23 (8.2%)
N3	4 (1.4%)
Pathologic grade	
Low grade	31 (11.1%)
High grade	243 (86.8%)
Non-urothelial carcinoma	5 (1.8%)
lymph node yield	8 (5-12)
Negative margin	270 (96.4%)
Positive margin	10 (3.6%)

BMI, Body Mass Index; ORC, open radical cystectomy; LRC, laparoscopic radical cystectomy; RARC, robot-assisted radical cystectomy; EBL, estimated blood loss, ΔHb, (preoperative hemoglobin – postoperative nadir hemoglobin)/preoperative Hb × 100.

### Assessment of complications

A total of 225 (80.36%) patients suffered from complications after operations, and the total number of all postoperative complications was 528. The detailed summary of categories, grading (according to CDC), number and proportion of all types of complications are depicted in **Table 2**. The three most common complication types were cardiac complications (40.71%), infectious complications (36.43%), and gastrointestinal complications (32.4%). Nine (3.2%) patients received reoperation after radical cystectomy and PLND, and wound dehiscence was the most common cause (**Supplementary Table 2**). Sixteen (5.7%) patients were unplanned readmitted after the primary operation, and the most frequent complication for readmission was ileus (**Supplementary Table 3**). No patient died within 90-d after radical cystectomy. While two patients died shortly after the operation (on day 94 and day 104). Four (1.43%) patients experienced intraoperative complications, all of which were rectal injury.

**Table 2:** Frequencies, proportions, therapeutic management, and grading of perioperative 90-d complications in patients who underwent radical cystectomy and pelvic lymph node dissection.

Comlications	Management	CDC grading	Number (proportion)
Gastrointestinal complications			90 (32.14%)
Paralytic ileus	Conservative; cessation of oral intake and i.v. fluid support	I	13 (4.64%)
	Indwelling of nasogastric tube	IIIa	33 (11.79%)
Mechanical intestinal obstruction	Surgery	IIIb	1 (0.36%)
Constipation	Conservative; laxatives	I	18 (6.43%)
Gastrointestinal bleeding	Conservative; clinical observation or diagnostic evaluation only	I	7 (2.50%)
	Blood transfusion	II	2 (0.71%)
Emesis	Conservative; antiemetics and i.v. fluid support	I	3 (1.07%)
Diarrhea	Conservative; antidiarrheals, i.v. fluid support, electrolytes	I	13 (4.64%)
Genitourinary complications			30 (10.71%)
Acute kidney injury	Conservative; i.v. fluid support, diuretics	I	22 (7.86%)
Hydronephrosis/ureteral obstruction (new onset)	Conservative; clinical observation or diagnostic evaluation only	I	6 (2.14%)
Urinary leak/urinoma	Surgery	IIIb	1 (0.36%)
Urinary retention	Conservative; clinical observation or diagnostic evaluation only, replacement of Foley catheter (neobladder/pouch)	I	1 (0.36%)
Cardiac complications			114 (40.71%)
Arrhythmia	Conservative; medical cardioversion	II	34 (12.14%)
Myocardial infarction	Coronary angiography and stent implantation, ICU	IVb	3 (1.07%)
Hypertension	Antihypertensives	II	47 (16.79%)
Heart failure	Conservative; Medical treatment	II	5 (1.79%)
	Medical treatment, ICU	IVa	1 (0.36%)
Angina (pectoris) or electrocardiogram changes	coronary vasodilating drugs	II	17 (6.07%)
Hypotension	Medical treatment	II	7 (2.50%)
Pulmonary complications			30 (10.71%)
Atelectasis	Continuous positive airway pressure, physiotherapy	II	4 (1.43%)
Pneumonia	Antibiotic therapy	II	6 (2.14%)
Respiratory distress/dyspnea	Oxygen, physiotherapy	I	11 (3.93%)
Pleural effusion	Conservative; clinical observation or diagnostic evaluation only	I	3 (1.07%)
Asthma attack	Medical treatment	II	2 (0.71%)
Respiratory failure	Medical treatment, ICU	IVa	4 (1.43%)
Neurological complications			21 (7.50%)
Peripheral neuropathy	Conservative; clinical observation or diagnostic evaluation only	I	3 (1.07%)
Cerebrovascular accident, transient ischemic attack	Medical treatment	II	1 (0.36%)
	Medical treatment, ICU	IVa	2 (0.71%)
Delirium/agitation	Antipsychotics	II	7 (2.50%)
Vertigo	Medical treatment	II	4 (1.43%)
Loss of consciousness/syncope	Conservative; clinical observation or diagnostic evaluation only	I	4 (1.43%)
Infectious complications			102 (36.43%)
Fever of unknown origin	Conservative; antipyretics, antibiotic treatment	II	59 (21.07%)
Asymptomatic bacteriuria	Conservative; no antibiotic treatment	I	1 (0.36%)
Urinary tract infection	Antibiotic treatment	II	13 (4.64%)
Abscess	Antibiotic treatment	II	1 (0.36%)
	Puncture drainage	IIIa	1 (0.36%)
	Incision and drainage + general anesthesia	IIIb	1 (0.36%)
Sepsis (SIRS in response to infectious process)	Antibiotic treatment, supportive care	II	19 (6.79%)
	Septic multiorgan dysfunction, ICU	IVb	4 (1.43%)
Pyelonephritis	Antibiotic treatment	II	1 (0.36%)
Gastroenteritis	Antibiotic treatment	II	1 (0.36%)
Cholecystitis	Antibiotic treatment	II	1 (0.36%)
Wound complications			25 (8.93%)
Wound fat liquefaction	Conservative; clinical observation or diagnostic evaluation only	I	12 (4.29%)
Wound infection (SSI)	Antibiotic treatment	II	4 (1.43%)
Wound dehiscence (fascia intact)	Conservative; clinical observation or diagnostic evaluation only, reinforced adhesive skin closure	I	4 (1.43%)
Fascial dehiscence/evisceration	Secondary surgical closure	IIIb	5 (1.79%)
Bleeding			48 (17.14%)
Anemia requiring transfusion	Blood transfusion	II	44 (15.71%)
Postoperative bleeding other than gastrointestinal	Blood transfusion/fibrinogen	II	3 (1.07%)
	surgical revision	IIIb	1 (0.36%)
Thromboembolic complications			15 (5.36%)
Deep vein thrombosis	Anticoagulation	II	14 (5.00%)
Pulmonary embolism	Anticoagulation	II	1 (0.36%)
Miscellaneous complications			53 (18.93%)
Dermatitis	Ointment	I	5 (1.79%)
Lymphocele	Conservative; clinical observation or diagnostic evaluation only	I	1 (0.36%)
Hypokalemia	Conservative; medical therapy	I	32 (11.43%)
Transaminase elevations	Conservative; medical therapy	II	15 (5.36%)
Intraoperative complications^a^			
Rectal injury	Revision surgery	–	4 (1.43%)

CDC, Clavien-Dindo classification; ICU, intensive care unit; i.v., intravenous.

a The CDC does not apply to intraoperative complications. Thus, no grading system was used.

### CDC and CCI

**Figure 1:**
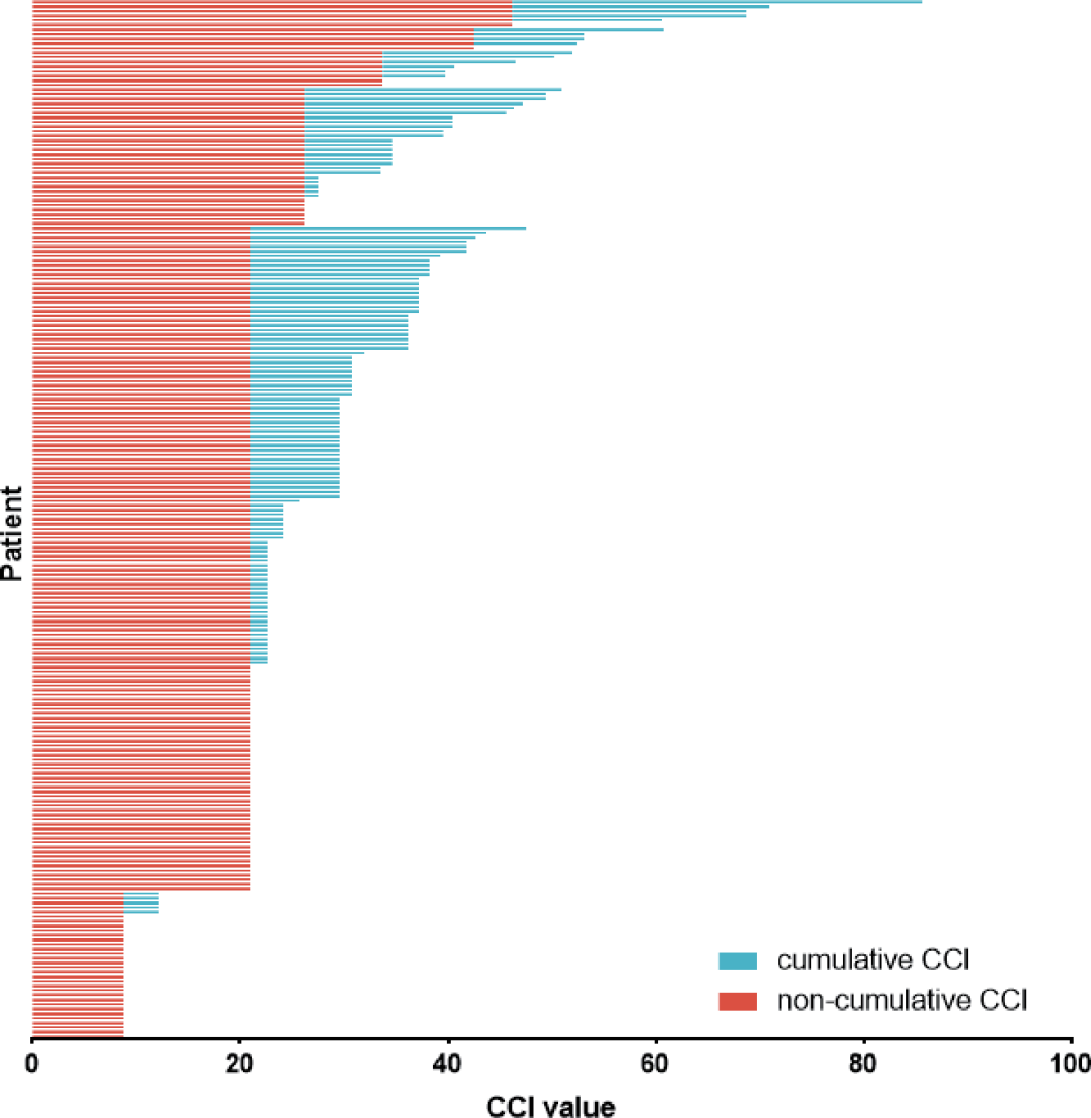
Non-cumulative CCI and cumulative CCI of patients with complications. CCI = Comprehensive Complication Index.

The cumulative CCI was calculated for each patient with postoperative complications. Patients with higher grades of CDC had a higher CCI (**Supplementary Figure 1A**, r = 0.879, *p* < 0.001). A total of 139 (49.6%) patients developed more than one complication after radical cystectomy and PLND. With increasing CDC grade, patients had a higher number of complications (**Supplementary Figure 1B**). With an increasing grade of CDC, more patients had a higher cumulative CCI compared with the non-cumulative CCI, which only considered the most severe complication (**Figure 1**). The above results indicate that the cumulative CCI was more representative of elderly patients’ burden of postoperative complications.

**Figure 2:**
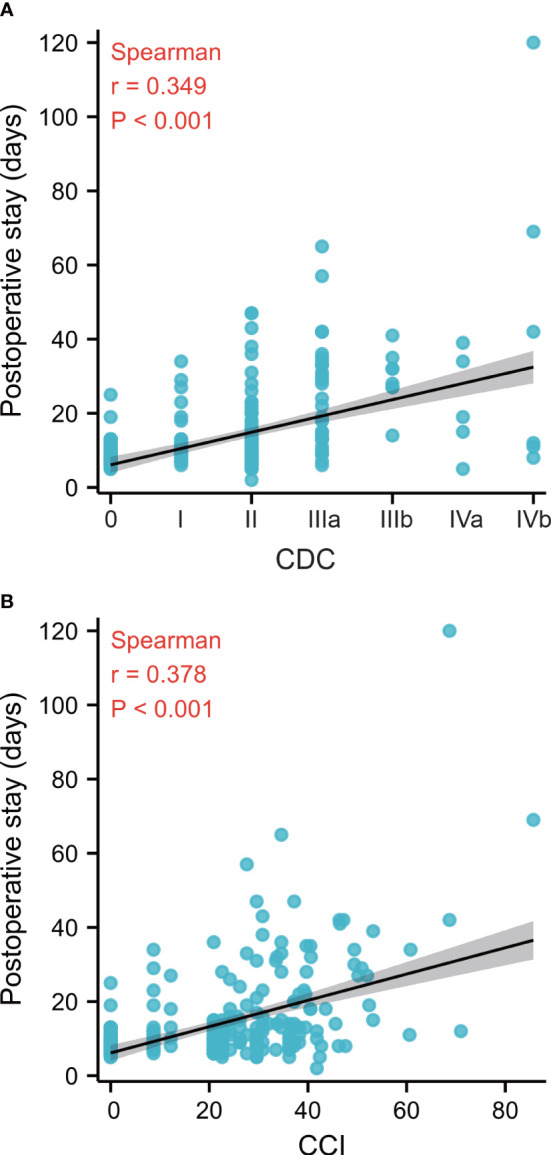
**(A)**: Correlation between CDC and postoperative stay (r = 0.349, p < 0.001). **(B)**: Correlation between CCI and postoperative stay (r = 0.378, p < 0.001). CDC, Clavien–Dindo classification, CCI, Comprehensive Complication Index. The correlations between variables were analyzed using the Spearman rank correlation coefficient.

The correlations of postoperative stay with cumulative CCI and CDC were analyzed (**Figure 2**). The cumulative CCI, taking into account all complications, had a more accurate prediction of postoperative stay than the CDC, considering only the most severe complications (r = 0.378, *p* < 0.001 vs. r = 0.349, *p* < 0.001).

In order to compared sensitivity of CCI and CDC to identify the difference of postoperative morbidity caused by treatment. The sample sizes were calculated when the presence of any CDC complication, presence of major CDC complication, and cumulative CCI were used for the primary endpoint. The assumptions for the fictive superiority trial are depicted in **Table 3**, and the need for sample size could significantly decrease when cumulative CCI was used for the primary endpoint.

**Table 3:** Sample size calculation for surgical RCTs using different measurements.

	Assumptions	Sample size
Presence of any CDC complication	30% relative risk reduction	55 patients per group
Presence of major CDC complication	30% relative risk reduction	2054 patients per group
CCI	Δ10 point, SD = 15.85	41 patients per group

RCTs, Randomized Controlled Trials; CDC, Clavien-Dindo classification; CCI, Comprehensive Complication Index.

### Risk factors for major complications

When CDC grade ≥IIIb was defined as the endpoint of major complications, Charlson comorbidity index ≥3 (OR = 6.34 [1.93-20.76], *p* = 0.002) and higher ΔHb (OR = 1.06 [1.01-1.10], *p* = 0.017) were identified as risk factors for major complications in elderly patients after radical cystectomy. While not only Charlson comorbidity index ≥3 (OR = 2.86 [1.19-6.86], *p* = 0.019) and higher ΔHb (OR = 1.06 [1.03-1.09], *p <*0.001) but also preoperative renal insufficiency (OR = 2.59 [1.02-6.62], *p* = 0.046) and higher time of operation (OR = 1.01 [1.00-1.01], *p* = 0.040) were associated with major complications, when the endpoint of that was referred to CCI ≥33.7 (**Table 4**).

**Table 4:** Logistic regression analysis of variables associated with major complication for all patients.

Variable	CDC grade ≥ IIIb vs. CDC grade ≤ IIIa			CCI ≥33.7 vs. CCI <33.7		
	OR	95% CI	*p*	OR	95% CI	*p*
Age (continuous)	0.96	0.81-1.14	0.653	1.07	0.97-1.17	0.183
Sex						
female	-	referent	-	-	referent	-
male	1.59	0.32-7.89	0.567	0.79	0.37-1.69	0.540
Charlson comorbidity index						
≤2	-	referent	-	-	referent	-
≥3	6.34	1.93-20.76	0.002	2.86	1.19-6.86	0.019
Preoperative renal insufficiency	3.39	0.83-13.85	0.090	2.59	1.02-6.62	0.046
Time of operation (continuous)	1.00	0.99-1.01	0.352	1.01	1.00-1.01	0.040
ΔHb	1.06	1.01-1.10	0.017	1.06	1.03-1.09	<0.001
Pathologic stage			0.156			0.957
Ta and Tis and T1	-	referent	-	-	referent	-
T2	0.18	0.03-0.91	0.038	0.90	0.41-1.98	0.793
T3	0.45	1.12-1.76	0.252	0.83	0.36-1.92	0.659
T4	1.01	0.22-4.74	0.988	0.76	0.25-2.25	0.614
Pathologic nodal stage						
N0	-	referent	-	-	referent	-
N+	0.36	0.06-2.31	0.280	1.75	0.69-4.44	0.238

CDC, Clavien-Dindo classification; CCI, Comprehensive Complication Index; ΔHb, (preoperative hemoglobin – postoperative nadir hemoglobin)/preoperative Hb × 100, CCI, 33.7 corresponds to CDC, IIIb.

## Discussion

In the present study, we assessed the 90-d complications of elderly patients after radical cystectomy and PLND by implementing the EAU guideline for reporting complications. The CDC and CCI were used and compared as the severity grading system for postoperative complications. Compared with CDC, cumulative CCI could represent the burden of complications more sensitively and presented stronger correlation with postoperative stay. When cumulative CCI was applied as the primary endpoint for a fictive superiority trial, the need for sample size would decrease. Furthermore, more risk factors for major complications were identified when CCI ≥33.7 was defined as the endpoint of major complications.

Previous studies have shown that there is large variation in reporting postoperative complications after radical cystectomy (2–7, 10), which is caused by the interobserver variability in defining and grading complications (16). Implementation of the EAU guidelines on reporting complications contributed to improving the accuracy and reliability of reporting and grading complications (9, 10). In our study, most patients (80.36%) suffered from at least one complication after radical cystectomy, which was higher than the majority of other studies (2–5). Standardized criteria could improve the quality of complication reporting and allow comparison of different treatment. Furthermore, through rigorous evaluation of clinical data, a greater number of complications would be detected, especially grade 1 and 2 complications (according to CDC). We found a total of 528 postoperative complications in our cohort, and almost half of the patients experienced more than one complication. Evaluation of the burden of complications based on CDC, which only considers the most severe complication, would be underestimated. We also found that 83 (29.6%) patients had an elevated CCI corresponding to CDC. The CCI was superior to the CDC when estimating the burden of morbidity after surgery, which has also been validated in other surgeries, such as gastric cancer surgery (17) and pancreatectomy (18).

In this study, we found that CCI exhibited a stronger correlation with postoperative stays than CDC, which was the same as previous studies of open radical cystectomy (13) and gastric cancer surgery (17). These results proved that the CCI grading system can be mirrored by the perioperative outcomes of patients more accurately. However, in the literature, for radical prostatectomy, partial nephrectomy (13) and endourological stone surgery (19), there was no superiority of the correlation between CCI and postoperative stay compared with CDC. The low incidence of complications (29.3% to 41.2%) and short hospital stay of these surgeries could explain this result of no superiority (13, 19). Nevertheless, the postoperative stay should not be the only parameter representing the perioperative outcomes. Further studies are encouraged to evaluate the correlations of grading systems of complications between objective and subjective perioperative characteristics, such as treatment cost, treatment satisfaction of patients and patients’ outcomes.

Slankamenac K. et al. first proved that the requirement of sample size was lower for CCI than traditional endpoints for the superiority trial on morbidity endpoints (20). In the present study, we also evaluated the sample size for a fictive superiority trial when different events were used as endpoints. The CCI could significantly reduce the need for sample size, especially compared with the presence of major CDC complications. This suggests that the use of CCI as the primary endpoint in future superiority trials could increase the feasibility and decrease the cost of the clinical trial.

When CCI ≥33.7 was defined as the endpoint of major complications, more risk factors were identified in the present study. To the best of our knowledge, there was no study reporting this superiority of sensitivity for exploring risk factors before. Preoperative renal insufficiency and time of operation were covered up when using conventional definition of major complications because of the small number of endpoint events and little effect on the endpoint, which may be prominent when there was a larger sample size. In another study with a larger sample size, preoperative renal insufficiency was indeed identified to be associated with major complications (21). The complications and management of patients with preoperative renal insufficiency are depicted in **Supplementary Table 4**. There were 31 patients with preoperative renal insufficiency, and 25 (80.6%) patients suffered from postoperative complications. The number of complications per patient for patients with preoperative renal insufficiency was 2.52, which was higher than that for patients with normal renal function. A greater number of complications could explain the upgrade of the CCI score.

Several limitations should be considered in this study. First, the retrospective design would cause information loss because of recall bias. It was definitely underestimated for postoperative complications, although we report a relative high morbidity of complications. Second, for intraoperative complications, only rectal injury was recorded in the case data, the fact and detail of that were not able to report. Moreover, the CDC could not apply to intraoperative complications, and further study should assess the intraoperative complications by using a grading system for urological surgery, such as the “Intraoperative Adverse Incident Classification” (22). Third, PLND is the main part of the surgery procedure, which could increase the time of operation (23) and was strongly associated with postoperative complications. From a statistical perspective, it was not feasible to enrolled patients received different surgical procedures. Thus, elderly patients who did not receive PLDN were excluded. Finally, the rate of complication would be overestimated, because some adverse events would happen even without attack of radical cystectomy. But in this study, the complications recorded were occurred during the period of the treatment of radical cystectomy, and were all related to the treatment of surgery.

## Conclusion

CCI, compared with CDC, is better for evaluating the severity of complications in elderly patients after radical cystectomy and PLND by implementing the EAU guideline for complication reporting.

## Data availability statement

The raw data supporting the conclusions of this article will be made available by the authors, without undue reservation.

## Ethics statement

The studies involving human participants were reviewed and approved by the clinical research ethics committee of Peking University First Hospital. Written informed consent for participation was not required for this study in accordance with the national legislation and the institutional requirements.

## Author contributions

All authors contributed to the study conception and design. Material preparation, data collection and analysis were performed by HWH, ZZ, HH, and MS. The first draft of the manuscript was written by HWH and HW. and all authors commented on previous versions of the manuscript. All authors read and approved the final manuscript.

## Funding

This study was supported by the National Natural Science Foundation of China (grant numbers: 81272829).

## Conflict of interest

The authors declare that the research was conducted in the absence of any commercial or financial relationships that could be construed as a potential conflict of interest.

## Publisher’s note

All claims expressed in this article are solely those of the authors and do not necessarily represent those of their affiliated organizations, or those of the publisher, the editors and the reviewers. Any product that may be evaluated in this article, or claim that may be made by its manufacturer, is not guaranteed or endorsed by the publisher.

## Abbreviations

PLND, pelvic lymph node dissection; CDC, Clavien–Dindo classification; CCI, Comprehensive Complication Index; EAU, European Association of Urology; EBL, estimated blood loss; SCr, serum creatinine.
