# Supplementary figures and images for: The comprehensive complication index is more sensitive than the Clavien–Dindo classification for grading complications in elderly patients after radical cystectomy and pelvic lymph node dissection: Implementing the European Association of Urology guideline
Source: Front Oncol. 2022 Oct 20;12:1002110. doi: 10.3389/fonc.2022.1002110 (PMC9631924; doi:10.3389/fonc.2022.1002110)

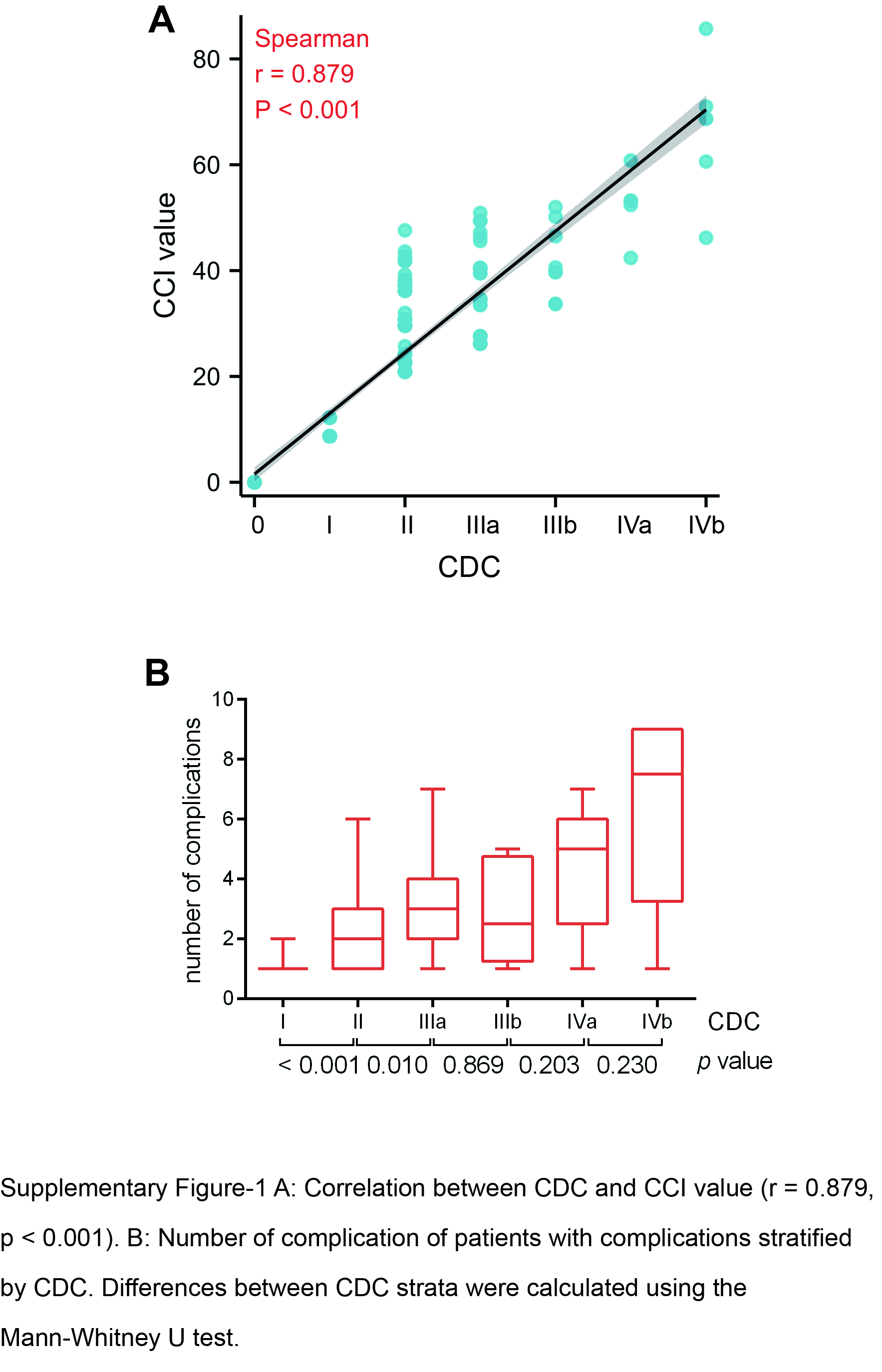

Supplement: Supplementary file 1 [file Image_1.tif]
